# Medical student self-reported confidence in obstetrics and gynaecology: development of a core clinical competencies document

**DOI:** 10.1186/1472-6920-13-62

**Published:** 2013-05-01

**Authors:** Kristen Pierides, Paul Duggan, Anna Chur-Hansen, Amaya Gilson

**Affiliations:** 1 Medical School, The University of Adelaide, Frome Rd, Adelaide, 5005, Australia; 2 Discipline of Obstetrics and Gynaecology, The University of Adelaide, Frome Rd, Adelaide, 5005, Australia; 3 Discipline of Psychiatry, The University of Adelaide, Frome Rd, Adelaide, 5005, Australia

**Keywords:** Clinical skills, Obstetrics and Gynaecology, Core competencies, Student evaluation

## Abstract

**Background:**

Clinical competencies in obstetrics and gynaecology have not been clearly defined for Australian medical students, the growing numbers of which may impact clinical teaching. Our aim was to administer and validate a competencies list, for self-evaluation by medical students of their confidence to manage common clinical tasks in obstetrics and gynaecology; to evaluate students’ views on course changes that may result from increasing class sizes.

**Methods:**

A draft list of competencies was peer-reviewed, and discussed at two student focus groups. The resultant list was administered as part of an 81 item online survey.

**Results:**

Sixty-eight percent (N = 172) of those eligible completed the survey. Most respondents (75.8%) agreed or strongly agreed that they felt confident and well equipped to recognise and manage most common and important obstetric and gynaecological conditions. Confidence was greater for women, and for those who received a higher assessment grade. Free-text data highlight reasons for lack of clinical experience that may impact perceived confidence.

**Conclusions:**

The document listing competencies for medical students and educators is useful for discussions around a national curriculum in obstetrics and gynaecology in medical schools, including the best methods of delivery, particularly in the context of increasing student numbers.

## Background

It is well established that the graduating medical practitioner must have knowledge and expertise in women’s health [1]. Whilst Competency Maps have been developed for specialist training [2], expected competencies have not been well defined for medical students. Efforts have been made in the United States to identify priority learning objectives in Obstetrics and Gynaecology [3]. In Australia, there has been an attempt to define a national core curriculum in Women’s Health [4]. However, a national curriculum for medical schools in Australia is lacking, in Obstetrics and Gynaecology as well as other specialty areas [5]. The Australian Junior Doctors’ Curriculum Framework [6] outlines the core knowledge and skills expected of a junior doctor as determined by the Postgraduate Medical Education Council, which does not include specialists in Obstetrics and Gynaecology. The competencies of this Framework broadly cover all medical disciplines. Arguably, the document is not detailed enough to be more than a guide to pre-graduation teaching in Obstetrics and Gynaecology.

Patients are having acute and shorter hospital stays and therefore finding quality clinical placements is becoming increasingly difficult [7]. An added pressure for many medical schools around the world are growing class sizes [8], a challenge that is not new, with the impact described as early as 1978 [9]. Furthermore, clinical placements are needed not only for medical students, but also for other health professional trainees, including nursing and midwifery students, meaning there may be competition for access to clinical exposure. In Australia, one strategy to increase opportunities for clinical exposure has been the inclusion of rural placements.

The University of Adelaide offers a 6-year undergraduate entry Medical program (the MBBS). The Year 5 Human Reproductive Health (HRH) course run by the Discipline of Obstetrics and Gynaecology is a mandatory 9-week component of the 5th year of the MBBS run over 4 terms in the academic year. The clinical component of teaching in this course involves allocation by roster of students to clinical placements in five metropolitan teaching hospitals and also a rural clinical school. The 2011 intake of approximately 150 students in Year 5 of the MBBS Program is to be progressively increased over the next several years, and is expected to peak at about 200 per annum. This has challenged us to consider ways to be more efficient in the delivery of the course and to identify new clinical placement opportunities. It is also helpful to consider what we are doing well and should continue, what we are doing that perhaps we should not be doing, and where we need to improve.

The primary aims of this research were to develop, validate and administer an on-line survey, for self-evaluation by senior medical students of their confidence to manage common clinical problems and tasks in Obstetrics and Gynaecology, following completion of the undergraduate Year 5 course in Human Reproductive Health (HRH) in the MBBS Program at the University of Adelaide. The primary objectives were to assist faculty to define the core clinical competencies in the course, to produce a list of clinical competencies in Obstetrics and Gynaecology by which students could self-assess their perceived confidence in these domains, and as a guide to teachers in the course.

The secondary aims were to seek opinions on the strengths and weaknesses, specifically, of clinical learning in the current course, to seek students’ views on possible reductions in clinical experience, and on alternative teaching methods, that may be required as a result resulting of increasing student numbers. The secondary objective was to assist faculty in its deliberations regarding course restructuring that will be required to meet the needs of additional students. Furthermore, we aimed to produce information that may be useful in informing a national curriculum in obstetrics and gynaecology.

Whilst there is not necessarily any correlation between competence and confidence [10], high levels of self-reported lack of confidence across a cohort may indicate areas of the curriculum which need to be examined more closely, including the reasons for this lack of confidence, which may be related to limited exposure in clinical settings.

## Methods

The University of Adelaide Human Research Ethics Committee granted approval for this study.

The Australian Junior Doctors’ Curriculum Framework (AJDF) [6] states junior doctors “should be able to appropriately assess patients presenting with common, important conditions including the accurate identification of symptoms/signs or problems and their differential diagnosis and then use that information to further manage the patient, consistent with their level of responsibility.” With reference to the expected competencies in the AJDF and our current curriculum, a draft survey was constructed seeking feedback about how well an individual felt that they had been prepared by their teaching to perform the tasks/procedures expected at intern level. We defined this to mean “When considering the answers, the expectation is that you are competent to perform to the level of an intern on the first day of his/her first attachment (e.g. to the emergency department)”. The draft survey was examined by 9 consultants in Obstetrics and Gynaecology and one Neonatologist, who were actively engaged in clinical practice and in teaching of medical students, and who provided comments and suggested additions and changes. This group also determined, where relevant, the faculty expectations of the level of performance that should be attained for individual tasks or scenarios. These expectations were “traffic light” colour-coded as follows: green = expected to function independently without direct supervision, orange = expected to be able to undertake the task under direct supervision only, red = expected to be able to describe the task only.

In order to further establish the face validity and acceptability of the survey instrument, students who had completed their Human Reproductive Health (HRH) rotation were asked to participate in a focus group conducted by a researcher with no involvement in medical school teaching. An advertisement was distributed to all Year 5 and 6 students, seeking their participation in one of two focus groups comprising 8 students, to discuss their reactions, experiences and attitudes about the clinical teaching they have received in HRH. We sought any students with a mixture of views such as those who liked or benefited and from those who disliked or didn’t benefit from the rotation. We sought volunteers with an Indigenous background, Rural background and International students, to get a broad mixture of views. Participants were asked to complete the survey online before attending the group. They were asked to discuss how well the survey reflected their experiences of teaching and learning in Obstetrics and Gynaecology, and particularly if any important skills or knowledge had been omitted. They also gave comments on any other items they felt should be included. Further, they were asked to speak freely about their perceptions of the Human Reproductive Health rotation. As an incentive to participate, all volunteers went into a draw for a $200 gift voucher from a major department store.

The data from the focus groups were transcribed and de-identified, and the survey modified in light of these data.

Focus groups were held in July 2011. The first group comprised 5 students (as 3 volunteers failed to attend). Three participants were women, one of whom was from a rural background. The second group comprised 7 students (with two volunteers failing to attend); five were women and none were from a rural background. Despite repeated calls during the recruitment phase, which lasted for 7 weeks, no Indigenous or International students volunteered for participation in a focus group. Focus group 1 (FG1) was of 60 minutes duration, FG2 70 minutes.

A number of structural changes to the survey were suggested during the focus groups, such as dividing sections more clearly and inserting a progress bar. It was ascertained from the focus groups that the survey took about 10 minutes to complete. Much discussion was peripheral to the survey and is not reported here. However, several themes of relevance were discussed: the usefulness of the survey as a teaching and learning tool; the impact of competition for clinical exposure on placements; and a perceived need for exposure to pregnant women, with a rejection of standardised patients and simulated learning as reasonable or sufficient substitutes.

The modified survey was then piloted, again modified and then placed online. All students in Years 5 (N = 153: 59.5% women) and 6 (N = 117: 56.4% women) who had been taught in the HRH course in 2010 or 2011 were asked to complete the voluntary, anonymous survey, via email requests and via several requests during lectures.

Free text responses were examined by the third author (ACH), and a content analysis performed by examining the themes apparent in the data and counting the number of times these themes were evident. The other three authors considered the resultant content analysis in relation to the raw data, for rigour.

The final survey comprised 5 demographic items, 7 items eliciting free comment, and 74 skills-related items requiring a response on a 5-point Likert scale (Strongly Agree to Strongly Disagree). Of these 74 items, 1 was a global rating item, 61 covered clinical skills in specific presentations, and 12 addressed domains of clinical care.

Statistical analysis was descriptive. A copy of the final survey can be requested from the second author (PD).

## Results

The survey was completed by 68% (n = 172) of the 270 medical students (58.1% women) invited to participate. Five surveys (2.9%) were incomplete. Fifty-nine percent (n = 102) of survey respondents were women. The 5-point Likert responses were trichotomised (agree and strongly agree = positive responses; neutral; disagree and strongly disagree = negative responses) and the proportion of positive responses described for each statement.

Twenty-two percent of respondents (n = 38) were interested in a career in Obstetrics and Gynaecology. The majority of students (75.8%, n = 125) agreed or strongly agreed with the statement that they felt confident and well equipped to recognise and manage most common and important obstetric and gynaecological conditions. However, 7.9% (n = 13) of students disagreed or strongly disagreed and 16.4% (n = 27) were undecided. Level of confidence was not associated with year level (5 or 6) or hospital location at which the HRH rotation was undertaken.

There was no relationship between having an interest in Obstetrics and Gynaecology and overall confidence to recognise and manage most common and important obstetric and gynaecological conditions. However the grades (A, B, C or D) that students received for their Obstetrics and Gynaecology course were associated with higher confidence (Fishers exact test, p = .001) with 88.6% of students receiving an A grade agreeing or strongly agreeing that they were confident. Men were significantly less confident than women in the management of common and important obstetric and gynaecological conditions (chi-square = 11.6, df = 2, p = .004).

**Figure 1:**
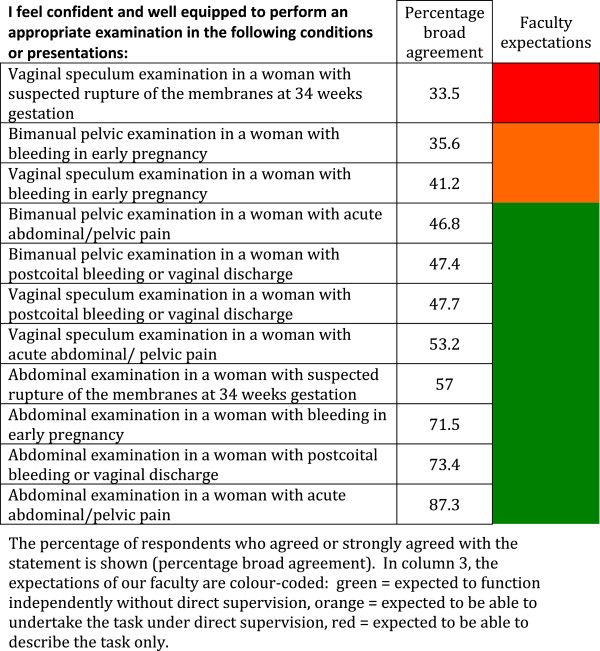
Student self perceived confidence in examination skills.

**Figure 2:**
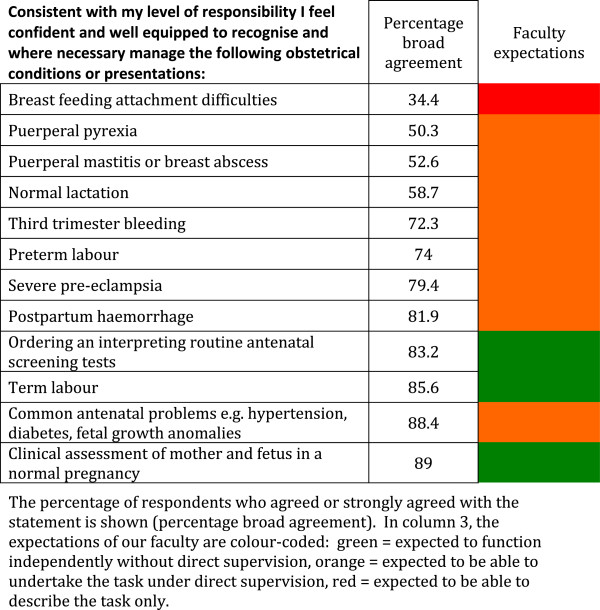
Student self perceived confidence in management of 12 common antenatal or postnatal scenarios.

The survey, as a template for a core curriculum document, assessed reported confidence in specific areas of Obstetrics and Gynaecology. The results are reported in 6 categories (Tables 1 and 2, Figures 1 and 2, Tables 3 and 4): (1) Meeting the requirements of the Australian Junior Doctors Framework, (2) History-taking skills, (3) Examination skills, (4) Management of obstetrics conditions, (5) Management of neonatal conditions, and (6) Procedures to be performed without direct supervision. Overall, perceived confidence ranged from 33.5% for conducting a vaginal speculum examination in a woman with suspected PPROM (a task our faculty determined students should be expected to be able to describe only) to 99.3% for measuring blood pressure in pregnancy (a task our faculty expected students to be able to undertake independently without direct supervision). We arbitrarily determined that confidence would be classified as “high” if 70% or more of students broadly agreed that they were confident in the task or procedure, moderate if 50-69% broadly agreed that they were confident in the task or procedure, and low if less than 50% felt confident in the task or procedure.

**Table 1:** **Student self perceived confidence in 12 clinical skills domains in the context of the expectations defined by the Australian Junior Doctor Framework**[6]

**I had sufficient clinical teaching to meet the requirements of the Australian Junior Doctor Framework (including the accurate identification of symptoms/signs or problems and their differential diagnosis and the use of that information to further manage the patient) in the following domains:**	**Percentage broad agreement**
Ward duties of resident medical staff in a gynaecology unit	37.7
Ward duties of resident medical staff in a maternity unit	44.2
Care of the term neonate	56.8
Common gynaecological surgical procedures	57.8
Common emergency gynaecological presentations	58.7
Common obstetrical surgical procedures	64.6
Postpartum care	65.2
Medical management of common gynaecological conditions	73.4
Common outpatient gynaecological presentations	73.6
Intrapartum care	75.5
Medical management of common obstetrical conditions	87.7
Antenatal care	93

The percentage of respondents who agreed or strongly agreed with the statement is shown (percentage broad agreement).

**Table 2:** Student self perceived confidence in history taking in emergency and outpatient presentations

**I feel confident and well equipped to take a history in the following emergency presentations:**	**Percentage broad agreement**
Abdominal pain in late pregnancy	82.1
Vaginal bleeding in late pregnancy	84
Vaginal bleeding in early pregnancy	88.3
Acute abdominal/pelvic pain in a young woman	93.3
**I feel confident and well equipped to take a history in the following conditions or presentations to an outpatient or GP clinic:**	
Violence or sexual abuse	40
Premenstrual tension	45.3
Chronic pelvic pain	60.9
Dyspareunia	61.7
Infertility	64.6
Vaginal prolapse	70.4
Unplanned pregnancy	72
PCOS/hyperandrogenism	73.1
Urinary incontinence	75.7
Vaginal discharge	77.3
Sexually transmitted infections	82.6
Abnormal menstruation	82.7
Amenorrhea	83.3
A request for contraception	83.8
Menopausal symptoms	86.9
Postmenopausal bleeding	87.6

The percentage of respondents who agreed or strongly agreed with the statement is shown (percentage broad agreement). The expectation of our faculty was that students should be competent in history taking in all of the presentations listed.

**Table 3:** Student self perceived confidence in management of common neonatal scenarios

**Consistent with my level of responsibility I feel confident and well equipped to recognise and manage the following conditions or presentations in the neonate:**	**Percentage broad agreement**
Jaundice	70.7
Hypoglycaemia	56.7
Parental counselling regarding routine neonatal screening tests	74.2

The percentage of respondents who agreed or strongly agreed with the statement is shown (percentage broad agreement). The expectation of our faculty was that students should be competent in all of the scenarios listed.

**Table 4:** Student self perceived confidence in the performance of 15 common clinical skills

**I feel confident and well equipped to undertake the following without direct supervision:**	**Percentage broad agreement**
Inspection of an episiotomy wound	37.1
Postnatal breast examination	39.2
Pelvic examination to assess uterine size, presence of mass, the adnexae and for tenderness and/or "cervical excitation"	50.6
Assisting a woman to give birth normally, including delivery of the placenta (assume this is an unplanned delivery with a cephalic presentation at term in an emergency department)	58.3
Taking an endocervical swab for chlamydia PCR testing	66
Taking a vaginal swab for culture and sensitivity	68.6
Taking a Pap smear	71.8
Inspection of a caesarean section wound	71.8
Locating the cervix using a vaginal speculum	73.7
Abdominal examination to assess abdominopelvic masses	79.4
Physical examination of a well term neonate (including examination of the hips)	81.4
Inserting a wide bore IV cannula	85.9
Measuring symphysial-fundal height in pregnancy	96.1
Checking for the presence of a fetal heart using a hand-held Doppler ultrasound probe	97.5
Measuring blood pressure in pregnancy	99.3

The percentage of respondents who agreed or strongly agreed with the statement is shown (percentage broad agreement). The expectation of our faculty was that students should be competent in all of the skills listed.

The category “Meeting the requirements of the Australian Junior Doctors Framework” contained 12 clinical skills domains (Table 1).

These domains, in the opinion of our faculty, broadly covered the clinical areas that junior doctors in Australia could reasonably be expected to be engaged in. We expected that students should be able to competently undertake the basic tasks within these domains “including the accurate identification of symptoms/signs or problems and their differential diagnosis and the use of that information to further manage the patient”. In two domains (encompassing ward duties of junior medical staff) student confidence was low (less than 45% of students reporting confidence) and in five domains (encompassing non-operative obstetrics and non-emergency gynaecology) students confidence was high (>70%). In the remaining domains, confidence was moderate (between 56-65%).

In the category “History-taking skills” (Table 2), the expectation of our faculty was that students should be competent in history taking in all of the presentations listed. Students reported high levels of confidence in history-taking in common emergency presentations (>82%).

However, confidence in history-taking skills was low (< 46%) in presentations of “violence and sexual abuse” and “premenstrual tension” and only moderate (< 70%) in presentations of “chronic pelvic pain”, “dyspareunia” and “infertility”.

In the category “Examination skills” (Figure 1), the expectation of our faculty was that students should be competent in 8 of the 11 presentations listed. Of these 8 presentations, confidence was high (>71%) for only 3. Confidence was low (< 48%) in bimanual and speculum examination in all but one of the clinical scenarios listed.

In the category “Management of obstetrics conditions” (Figure 2), 12 common clinical scenarios were presented, of which the faculty expected students to be competent to manage independently only 3. In all 3 of these scenarios, students reported high levels of confidence (>83%). Confidence was lowest in the 4 lactation and puerperal scenarios (34.4-58.7%).

In the category “Management of neonatal conditions” our faculty expected students to be competent in the 3 scenarios listed (Table 3).

Student confidence was high (>70%) for two of the 3 neonatal scenarios and moderate (56.7%) for recognition and management of neonatal hypoglycaemia.

In the category “Procedures to be performed without direct supervision”, 15 procedures were listed and students were expected by our faculty to be competent to perform independently all procedures listed (Table 4). Student confidence was high (>70%) for 9 procedures, moderate for 4 procedures (50-69%) and low (< 40%) for 2 procedures (both puerperal procedures – breast examination and inspection of an episiotomy wound).

Of the free text responses, one-hundred and twenty-five (73%) participants responded to the query “What is your overall opinion of the adequacy of clinical teaching in HRH, such as live patient encounters in outpatients, delivery suite, wards and theatres?” Overall, comments were positive. Where criticisms were made, they were related to lack of exposure due to midwifery students taking precedence over medical students or midwives excluding medical students (15 comments), and a lack of “hands-on” experience (10 comments), including with deliveries (7 comments), being excluded because of gender (3 comments from men), and a lack of theatre experience (8 comments). Participants were asked whether reduction of teaching time in HRH would be a viable way in which to accommodate increasing student numbers: 69% (N = 119) of students commented, with 89 (75%) students rejecting this option, 15 (12.5%) seeing pros and cons, and 15 (12.5%) agreeing that reduction in teaching time would be an appropriate solution. Participants were asked to comment on the idea of replacing clinical teaching with standardised patients (SPs) and anatomical models in order to accommodate increased student numbers. Of the 118 (68%) students who commented, 43 (35%) agreed, 40 (34%) disagreed and 33 (28%) thought that both clinical exposure and supplementary teaching with SPs and anatomical models was the preferred option (2 comments were not relevant to the query).

## Discussion

The primary aims of this research were to develop, validate and administer an on-line survey, for self-evaluation by senior medical students of their confidence to manage common clinical problems and tasks in Obstetrics and Gynaecology. The document that was produced, was based on our present curriculum and with reference to the Australian Junior Doctors’ Curriculum Framework, ^6^ and then validated through expert opinion and feedback from students during focus groups, proved useful for identifying areas of perceived strength and weakness. The document has been used by our faculty in its review of the core clinical competencies in the course, and may serve as a useful basis for the development of national core clinical competencies in Obstetrics and Gynaecology. Provision of a list of expected core competencies at the beginning of a rotation may be beneficial so that students can review these and optimise their opportunity for clinical experience. Additionally, medical educators and clinicians can refer to the competencies when designing rotations, creating a curriculum, and when formulating assessments. This approach is commonly used in medical education [11,12].

The survey was intended to be globally comprehensive in relation to clinical skills and the results were expected be useful as a guide to faculty and students. A professorial group in the USA has identified 267 priority learning objectives in obstetrics and gynaecology [3], which we felt was unmanageably large. To keep the survey to a manageable size, some compromises were made. For example, although dating of pregnancy is a core clinical skill, it was not included in the survey questions, as it is a key requirement in the management of several of the included conditions.

Whilst the majority of the participants in this study reported good perceived overall confidence to recognise and manage most common and important obstetric and gynaecological conditions, it is notable that around one quarter of the sample were unsure or were clear that they were not confident. Although students are very confident taking a history, performing an abdominal examination, and in performing a speculum examination to take a Pap smear or cervical swab, they are significantly less confident with bimanual and speculum examination in other circumstances (e.g. suspected preterm premature rupture of the membranes - PPROM). It is interesting to note that students feel confident in taking Pap smears, as it has become difficult to provide students with opportunities to perform vaginal and speculum examinations in clinics. However, students are taught by ‘well-women’ teaching associates in vaginal and speculum examination, and we provide pelvic models for practising Pap smears and taking cervical swabs, which are also used in assessment. We have included competence in the performance of a Pap smear and cervical swab in our core competencies, because in either case proper identification of the cervix is required and this skill may be required of a junior doctor in some circumstances, e.g. suspected pelvic inflammatory disease presenting to an emergency department.

Somewhat concerning was the low confidence reported in the ability to manage an uncomplicated birth in the emergency department, performance of ward duties of resident medical staff in maternity and gynaecology units, and the management of common presentations such as normal lactation, breast abscess and mastitis. It was noted that the areas in which students reported low confidence were mostly related to the social taboos of genitals and breast. Students also expressed low confidence in the area of domestic violence and sexual abuse. Reasons for low confidence in these two latter areas cannot be ascertained in the data: however, lower confidence in some areas may be attributed to less exposure, as evidenced by the open-ended comments provided by participants. Challenges to clinical exposure related to interprofessional challenges related to midwifery students and midwives and a lack of “hands-on” experience with deliveries, in theatre, or because of male gender. The relationship between gender and confidence may in part be due to this reduced opportunity for exposure in clinical settings for men, caused by being asked to leave by midwives or by patients who feel uncomfortable with male students. It is also possible that women’s greater confidence can be attributed to identification with women’s health issues [13], although this can only be speculation.

Participants did not specifically identify increases in student numbers as problematic, but as students do not have increases within their cohorts (but rather, increases are successive over years), this is an issue that is very real to educators but may be less apparent to students.

Survey data showed that the reactions towards standardised patients as a teaching tool to increase confidence in core competencies was deemed by the majority to either be either acceptable, or acceptable as supplementary learning, provided that clinical exposure was also provided. Standardised patients and anatomical models have been repeatedly shown in the medical education literature to be a valuable adjunct to teaching [14] but should not be relied upon as a replacement for experience with real patients and clinical encounters.

A potential weakness of this study is that it is a measure of perception rather than an actual assessment of skills. Research is equivocal regarding the validity of self-evaluation and actual ability [15]. Further research into the perceived and actual competence of students could be guided by consideration of specific competencies. As a potential national document, further investigation around Australia of the role of class sizes and location of the medical school as well as other factors, on perceptions of confidence in Obstetrics and Gynaecology would be valuable for further refinement and confirmation of the validity of this tool for medical education.

## Conclusions

A document that lists core competencies for medical students and educators to utilise is a valuable tool. Further development of this tool would be useful, and may assist in discussions around a national curriculum in obstetrics and gynaecology in medical schools, including the best methods of delivery, particularly in the context of increasing student numbers.

## Competing interests

The authors declare they have no competing interests.

## Authors’ contributions

KP participated in the design of the study, distributed and collated the main survey and contributed to data analysis and interpretation and to the drafting of the manuscript. PD participated in the design of the study, contributed to data analysis and interpretation and to the drafting of the manuscript. AC-H participated in the design of the study, contributed to data analysis and interpretation and to the drafting of the manuscript. AG participated in the design of the study, conducted the focus groups, contributed to data analysis and interpretation and to the drafting of the manuscript. All authors read and approved the final manuscript.

## Pre-publication history

The pre-publication history for this paper can be accessed here:

http://www.biomedcentral.com/1472-6920/13/62/prepub
